# What Have Failed, Interrupted, and Withdrawn Antibody Therapies in Multiple Sclerosis Taught Us?

**DOI:** 10.1007/s13311-022-01246-3

**Published:** 2022-07-06

**Authors:** Julia Krämer, Heinz Wiendl

**Affiliations:** Department of Neurology With Institute of Translational Neurology, University Hospital Münster, Albert-Schweitzer-Campus 1, Building A1, 48149 Muenster, Germany

**Keywords:** Clinical trials, Drug development, Monoclonal antibodies, Antibody therapy, Failure, Multiple sclerosis

## Abstract

In the past two decades, monoclonal antibodies (mAbs) have revolutionized the treatment of multiple sclerosis (MS). However, a remarkable number of mAbs failed due to negative study results were withdrawn because of unexpected serious adverse events (SAEs) or due to studies being halted for other reasons. While trials with positive outcomes are usually published in prestigious journals, negative trials are merely published as abstracts or not at all. This review summarizes MS mAbs that have either failed in phase II–III trials, have been interrupted for various reasons, or withdrawn from the market since 2015. The main conclusions that can be drawn from these 'negative' experiences are as follows. mAbs that have been proven to be safe in other autoimmune conditions, will not have the same safety profile in MS due to immunopathogenetic differences in these diseases (e.g., daclizumab). Identification of SAEs in clinical trials is difficult highlighting the importance of phase IV studies. Memory B cells are central players in MS immunopathogenesis (e.g., tabalumab). The pathophysiological mechanisms of disease progression are independent of leukocyte 'outside-in' traffic which drives relapses in MS. Therefore, therapies for progressive MS must be able to sufficiently cross the blood–brain barrier. Sufficiently long trial duration and multicomponent outcome measures are important for clinical studies in progressive MS. The success of trials on remyelination-promoting therapies mainly depends on the sufficient high dose of mAb, the optimal readout for ‘proof of concept’, time of treatment initiation, and appropriate selection of patients. Failed strategies are highly important to better understand assumed immunopathophysiological mechanisms and optimizing future trial designs.

**Supplementary Information:**

The online version contains supplementary material available at 10.1007/s13311-022-01246-3.

## Introduction

The treatment landscape of multiple sclerosis (MS) has expanded very rapidly during the past 25 years, with 18 different disease modifying therapies (DMTs) currently available [1, 2]. Among these, monoclonal antibodies (mAbs) have gained particular interest and revolutionized the treatment of relapsing and, very recently, progressive MS, both with respect to efficacy and target specificity [1–6].

Natalizumab was the first mAb to be approved by the US Food and Drug Administration (FDA) for the treatment of MS in 2004, followed by alemtuzumab in 2014, ocrelizumab in 2017, and most recently ofatumumab in 2021. Despite the rapid progress in development of DMTs, the therapeutic landscape of highly active MS is far from complete as there is a particular dearth of immunotherapies indicated for secondary and primary progressive MS (SPMS, PPMS).

Even if the clinical trial success rates in MS surpass those of other disease areas by almost three times, only 27% of developed MS drugs reach approval [7]. Despite rational pathophysiological concepts, impressive data from animal models, and promising results of phase I/II studies, numerous promising candidates failed in clinical studies [8–15]. The reasons for their failure are diverse. Sometimes findings acquired from animal models were not transferable to humans; in other cases, poor study design with inappropriate study endpoints and insufficient follow-up durations obscured potential benefits of the investigated drug, or unexpected safety issues and risks led to premature study termination [16, 17]. While clinical studies with positive outcomes are usually published in prestigious journals, many negative trials are merely published as abstracts or not at all [12]. Sometimes, only the disappearance of a drug from a potential manufacturer’s portfolio indicates its ineffectiveness.

It is, however, highly important to critically analyse negative study results since failed trials add to our growing understanding of human MS immunopathogenesis and can provide valuable information how future studies and outcome measures should be designed [8, 10, 13, 17, 18]. Thus, ‘misses’ are at least as important to the science as the ‘hits’ and should therefore be available for the MS community [13, 18].

This review focuses on mAb therapies for relapsing and progressive MS that have been trialled in phase II–III studies since 2015 and that either failed to meet the primary study endpoint (pSE) or showed unexpected serious adverse events (SAEs) leading to cancellation of further studies or withdrawal from the world-wide market.

After describing the pathophysiological background, we present the design and results of clinical trials; explore potential reasons for failure, interruption, or withdrawn of mAbs; and discuss potential implications for our current understanding of MS pathogenesis and future trials on MS.

## Methods

To identify relevant articles published between 01 January 2015 and 30 November 2021, we conducted a MEDLINE search using the Medical Subject Headings (MeSH) terms ‘multiple sclerosis’, ‘therapy’, ‘antibody therapy’ or ‘antibody treatment’, ‘trial’, ‘failure’, ‘interruption’, and ‘withdrawn’. Since failed trials are often published as abstracts only or not at all, we additionally searched for relevant studies sourced from international conferences (Annual Meeting of the American Academy of Neurology [AAN], European/Americas Committee for Treatment and Research in Multiple Sclerosis [ECTRIMS/ACTRIMS]), and consultation of national and international registries for clinical trials (US National Library of Medicine; clinicaltrials.gov; European Union Drug Regulating Authorities Clinical Trials Database [EudraCT]). Table 1 gives an overview over failed phase II and III trials of discussed mAbs in relapsing and progressive MS.

**Table 1:** Overview of failed phase II and III trials in multiple sclerosis therapy over the last 5 years

**Drug(s)**	**(Assumed) mechanism of action**	**Phase**	**Study design**	**Subjects**	**Primary endpoints**	**Time frame**	**Study completion**	**Comment**	**NCT number/reference**
**Failed mAbs in relapsing multiple sclerosis**									
**B cell targeting therapies**									
Tabalumab (LY2127399)	Depletion of naïve/mature B cells and antibody formation	II	Double-blind, randomized, placebo-controlled, parallel-group, dose-ranging	245 RRMS	GELs	73 wk	2018	The missing treatment effect is probably due to missing depletion of memory B cells by tabalumab	NCT00882999 [77] *
Lanalumab (VAY736)		II	Partially-blind, randomized, placebo-controlled, parallel-group	96 RRMS	GELs	16 wk	2018	Study recruitment was terminated based on strategic considerations after enrolment of 8 patients	NCT02038049 *
Belimumab		II	Open-label, randomized, parallel-group	40 RRMS	Pneumococcal vaccine antibody response; AEs; SAEs	36 mo	Ongoing		NCT04767698
**Cytokine-directed therapies**									
Secukinumab (AIN457)	Prevention of production of pro-inflammatory mediators; Prevention of microglial and neutrophil recruitment; Promotion of remyelination and neural repair	II	Double-blind, randomized, placebo-controlled	73 RRMS	CUAL	24 wk	2012	Imbalances at baseline between groups and a subgroup of highly active patients in the secukinumab group; Evidence for a ‘delayed’ therapeutic effect	NCT01051817 [98]
		II	Open-label extension, randomized, placebo-controlled, parallel-group	39 RRMS	AEs	97 wk	2014	Further enrolment was stopped. This decision was not related to safety or tolerability concerns	NCT01433250 *
		II	Double-blind, randomized, placebo-controlled, parallel-group, dose-ranging	380 RMS	GELs	24 wk	2014	Early termination after inclusion of 28 patients based on development of CJM112	NCT01874340 [99]
CJM112		II	Double-blind, randomized	360 RRMS	GELs	24 wk		Not executed	[100]
**Antiviral approaches**									
Temelimab (GnbAC1)	Inhibition of immune activation, inflammation, and neurodegeneration, and promotion of remyelination	IIb	Double-blind, randomized, placebo-controlled	270 RRMS	GELs	48 wk	2018	No significant anti-inflammatory effect but a possible dose-dependent neuroprotective effect at the highest dose of 18 mg/kg	NCT02782858 [116]
		II	Long-term extension study	220 RRMS	Long term safety	48 wk	2018		NCT03239860 [116]
		IIa	Double-blind, randomized, placebo-controlled	41 RMS	AEs	48 wk	Active, not recruiting		NCT04480307
**Blocking lymphocyte trafficking**									
Vatelizumab	Preventing activated lymphocytes from binding to collagen fibres that build up in inflammatory sites	IIa/b	Double-blind, randomized, placebo-controlled	112 RRMS	GELs	108 wk	2016	Study discontinued based on planned interim analysis of the primary endpoint. Not linked to any safety concern	NCT02222948 [120]
**Neuroregenerative approaches**									
Opicinumab (anti LINGO-1)	Facilitation of oligodendrocyte differentiation and survival, axonal regeneration, and neuronal survival and regeneration, remyelination and functional recovery	II	Double-blind, randomized, placebo-controlled	82 AON	P100 latency using FF-VEP	32 wk	2014	Opicinumab treatment significantly ameliorated P100 latency at week 32 compared to placebo	NCT01721161 [137]
		II	Double-blind, randomized, placebo-controlled	330 RRMS 89 SPMS	CDI measured by EDSS, T25FW, 9HPT, PASAT-3	72 wk	2016	Weak evidence for CDI with 10 mg/kg and some improvement with 30 mg/kg opicinumab	NCT01864148 [142]
		II	Double-blind, randomized, placebo-controlled with optional open-label extension	263 RRMS/SPMS	Overall response score, an integrated assessment of CDI and CDP based on EDSS, T25FW, 9HPT / AEs, SAEs	72 wk/96 wk	2021	The decision to discontinue the open-label extension study was not based on safety concerns	NCT03222973 [143]

**Failed mAbs in progressive multiple sclerosis with otherwise approved and working compounds**									
**Blocking lymphocyte trafficking**									
Natalizumab	Blocking transmigration of B and T lymphocytes, macrophages, and dendritic cells into the CNS, and decreasing cerebral microglial activation	III	Double-blind, randomized, placebo-controlled with optional open-label extension	889 SPMS	Multicomponent measure of CDP (EDSS T25FWT 9HPT)/AEs, SAEs	96 wk/218 wk	2016	As predicted by the length-dependent axonopathy hypothesis, no effect on lower limb (EDSS and T25FW), but on upper-limb function (9HPT)	NCT01416181 [160]
Rituximab	CDC > ADCC	I/II	Double-blind, randomized, placebo-controlled	27 SPMS	CSF CXCL13 CSF BAFF	12 mo	2015	Incomplete depletion of B cells in CSF and CNS tissue	NCT01212094 [180]
		II/III	Double-blind, randomized, placebo-controlled	439 PPMS	12-week CDP	96 wk	2007	Significant effect of rituximab on pSE in patients younger than 51 years and/or with GELs	NCT00087529 [176]
		II	Open-label, randomized, placebo-controlled, parallel-group	10 PMS	Change in osteopontin level in CSF	12 mo	2019	Depletion of B cells in serum, but not in CSF	NCT02545959 [182]
		II/III	Open-label, randomized, parallel-group	84 SPMS	EDSS	12 mo	2019	Imbalances at baseline between groups	NCT03315923 [184]

*ADCC* antibody-dependent cellular cytotoxicity, *AE* adverse events, *9HPT* 9-hole peg test, *ARR* annualized relapse rate, *CDC* complement-dependent cytotoxicity, *CDI* confirmed disability improvement, *CDP* confirmed disability progression, *CUAL* combined unique active lesions defined as new gadolinium-enhancing lesions and new/enlarging T2-weighted lesions without double counting, *EDSS* Expanded Disability Status Scale, *FF-VEP* full-field visual evoked potential, *GELs* gadolinium-enhancing lesions, *mo* months, *pSE* primary study endpoint, *PMS* progressive multiple sclerosis, *PPMS* primary progressive multiple sclerosis, RMS relapsing multiple sclerosis, *RRMS* relapsing–remitting multiple sclerosis, *SAEs* serious adverse events, *SPMS* secondary progressive multiple sclerosis, *T25FWT* timed 25-foot walking test, *wk* weeks

*For studies marked with an asterisk, full publication is pending (last check 18.01.2021) and preliminary results from conferences or announcement press releases are reported. Clinical trials have been searched on https://clinicaltrials.gov, last accessed date 18.01.2021

## Failed mAbs in Relapsing Multiple Sclerosis

### T Cell Targeting mAbs

MS has traditionally been considered the ‘classical’ prototype of a T cell-mediated autoimmune disease [19]. Even if this view is considered no longer correct and involvement of nearly all cell types of the immune system was shown [20, 21], T cells still play a central role in the MS pathophysiology [22]. This view is based not only on data from extensive studies in animal models, but also accumulation of considerable numbers of T cells in inflammatory MS lesions [23] and data from different genome-wide susceptibility studies [24]. Many of the currently approved DMTs for MS target T lymphocytes, either by immune modulation (e.g., interferons), by depleting immune cell populations involving T cells (e.g., alemtuzumab), or by selective inhibition of distinct molecular pathways in order to sequester leucocytes (e.g., natalizumab) [22].

#### Daclizumab

##### Background

Daclizumab is a humanized immunoglobulin G1 (IgG1) mAb that reversibly binds the so-called Tac epitope (binding site for IL-2) on the α-subunit (CD25) of the high-affinity IL-2 receptor (IL-2R-HA), which is highly expressed on activated T lymphocytes and regulatory T (Tregs) cells [25, 26]. This blockade inhibits the proliferation of activated T-lymphocytes and their cytokine secretion, apoptosis of effector T cells, and early T cell activation through blockade of IL-2 transpresentation by dendritic cells. By reducing the IL-2 consumption by activated T cells, daclizumab increases the IL-2 bioavailability allowing for greater interaction with the intermediate-affinity IL-2R, and hereby driving the expansion and stimulation of immune regulatory CD56bright natural killer (NK) cells. These cells destroy activated CD4^+^ and CD8^+^ T cells, activated macrophages, dendritic cells, and immature microglia; reduce NK cell death; and further increase NK cell cytotoxicity. Moreover, it reduces the expansion of intrathecal pro-inflammatory lymphoid tissue-inducer cells, and indirectly reduces the formation of meningeal lymphoid follicle-like structures [27–29].

##### Studies

Daclizumab beta (Zinbryta™) was approved in 2016 by the FDA and European Medicines Agency (EMA) based on findings from two large phase IIb (CHOICE and SELECT) [30, 31] and one phase III (DECIDE) [32] study. SELECT was followed by the extension studies SELECTION [33] and SELECTED [34], while patients of the DECIDE were followed in the EXTEND study [35].

In CHOICE (NCT00109161), add-on subcutaneous high-dose daclizumab (2 mg/kg every 2 weeks, *n* = 75) could significantly reduce the number of new/enlarged gadolinium-enhanced lesions (GELs) (= pSE) in MS patients (92% relapsing–remitting MS (RRMS), 8% SPMS) with active disease despite treatment with interferon beta (IFNβ) compared to IFNβ alone (*n* = 77) and add-on low dose daclizumab (1 mg/kg every 4 weeks; *n* = 78) [31]. After this study, the chemical structure of daclizumab was modified to reduce the antibody-dependent cellular cytotoxicity (ADCC) and complement fixation [36] and called daclizumab high-yield process (DAC HYP).

In SELECT (NCT00390221), both DAC-HYP 150 mg (*n* = 208) and 300 mg (*n* = 209) significantly reduced the annualized relapse rate (ARR) (= pSE), the mean number of new GELs and T2 lesions, and the 12-week confirmed disability progression (CDP) compared to placebo (*n* = 204) [30]. 91% patients who completed SELECT enrolled into the 1-year extension trial SELECTION (NCT00870740) where those patients who received placebo in SELECT (*n* = 170) were randomized 1:1 to DAC-HYP 150 mg or 300 mg and those originally treated with DAC-HYP were randomized (1:1) either to continue prior treatment (*n* = 173) or to undergo a washout period of 20 weeks followed by reinitiation of their original dose (*n* = 174) [33]. The pSE was the safety and immunogenicity of DAC-HYP. Overall, frequencies of AEs and SAEs were similar between the treatment initiation and continuous treatment groups [33]. 90% (410/455) participants who completed SELECT and SELECTION received DAC-HYP 150 mg s.c. every 4 weeks for up to 6 years in the open-label extension study SELECTED (NCT01051349) (69% for > 3 years, 39% for > 4 years, and 9% for > 5 years) [34]. The efficacy of DAC-HYP on clinical and radiological disease activity was maintained throughout the study for up to ~ 8 years [34].

In DECIDE (NCT01064401), 1841 patients with active RRMS were randomized (1:1) to either 150 mg DAC-HYP or intramuscular IFNβ-1a for 96–144 weeks [32]. DAC-HYP significantly reduced the ARR (= pSE), the number of new GELs and T2 lesions, the brain atrophy, and the risk of increased 48-week CDP [32].

In EXTEND (NCT01797965), all participants who received either DAC-HYP (*n* = 606) or IFNβ-1a (*n* = 597) in DECIDE received DAC-HYP 150 mg for up to 5 years, followed by 24 weeks of post-dosing follow-up [35]. In the continuous DAC-HYP group, more participants were relapse free and fewer had experienced 24-week CDP than those who switched from IFNβ-1a in DECIDE to DAC-HYP in EXTEND [35].

##### Adverse Events

The rate of occurrence of AEs under DAC-HYP has been similar both to placebo and to IFNβ-1a [30, 32]. A pooled analysis of AEs from the pivotal studies of DAC-HYP and their extension studies, which encompassed 2236 patients (150 mg DAC-HYP: *n* = 1943; 300 mg DAC-HYP: *n* = 293) with 5214 patient-years of daclizumab exposure, demonstrated that the incidences of AEs (evaluated by 6-month intervals) remained stable over the 6.5 years of maximum follow-up [37]. Overall, most AEs were either of mild (*n* = 546; 24%) or moderate (*n* = 1080; 48%) severity, and 13% of patients (*n* = 299) under DAC-HYP had to discontinue treatment owing to an AEs other than MS relapse [37]. It is, however, important to note that comparator data was not included owing to heterogeneity across studies.

The incidence of SAEs that were reported with DAC-HYP in the clinical trials was higher compared to placebo and IFNβ-1a [30, 32–35]. Potentially serious infections, cutaneous reactions, and liver abnormalities were already described in the pivotal studies SELECT and DECIDE under treatment with 150 mg DAC-HYP. The extension studies additionally revealed serious inflammatory syndromes and lymphadenopathy that were previously not apparent.

Serious infections were more common in the 150 mg DAC-HYP groups (3% in SELECT and SELECTION, 4% in DECIDE, 6% in SELECTED, and 5% in EXTEND) compared to placebo (0%) and IFNβ-1a (2%) [30, 32–35]. In general, infections were managed in a conventional manner and for the most part did not require the suspension of DAC-HYP.

A variety of cutaneous AEs, mainly rash and eczema, occurred in up to one-third of patients, were mostly mild to moderate in intensity and resolved either spontaneously or after standard interventions [38]. Serious cutaneous events (e.g., exfoliative rash or dermatitis, toxic skin eruption, drug reaction with eosinophilia, and systemic symptoms (DRESS) syndrome) developed in < 1 to 2% under 150 mg DAC-HYP in the pivotal studies and up to 4% in the extension studies [30, 34, 35].

Elevations > 5 times the upper limit of normal (ULN) were more frequent under 150 mg DAC-HYP (4% in SELECT, 1% in SELECTION, 6% in DECIDE, 9% in SELECTED, and 8% in EXTEND) compared to placebo (< 1%) and IFNβ-1a (3%) [30, 32–35]. These increases were evenly distributed over time during treatment with DAC-HYP, self-limited, or could be successfully managed by treatment discontinuation and/or treatment with cortisone, and tended not to recur with continued treatment [27, 32, 38].

After one case of fatal liver failure due to hepatitis (in the washout and 300 mg DAC-HYP reinitiation group) in the SELECTION trial [33] as well as four cases of serious liver injury (confounded by concomitant drugs associated with liver toxicity) in EXTEND [35], a benefit-risk reassessment procedure was initiated in the European Union. This resulted in transitional safety regulations for treatment with daclizumab and licensing restriction to those adult patients who have had an inadequate response to at least two previous DMTs and for whom treatment with any other DMT was contraindicated or otherwise unsuitable (EMA/707022/2017).

Across all clinical studies, immune-mediated disorders occurred in 28% of patients on DAC-HYP, including lymphadenopathy and skin reactions [27, 39]. Immune-mediated SAEs developed in three patients in the SELECT trial under 300 mg daclizumab (thyroiditis, Crohn’s disease, hypersensitivity) [30], in five patients in the SELECTION trial (hepatitis, Graves’ disease, glomerulonephritis, and two cases of ulcerative colitis) [33], and in eight patients in SELECTED (two cases of hepatitis and uveitis, one case of vitiligo, morphoea, rheumatoid arthritis, and Sjögren’s syndrome) [34]. Surprisingly, no immune-mediated condition secondary to daclizumab treatment was reported in the DECIDE trial [32]. In EXTEND, 15 patients developed immune-mediated SAEs, of which three were encephalitis [35].

Following another case of fatal liver failure [40] and 12 reports of serious inflammatory brain disorders, including encephalitis and meningoencephalitis, three of them with fatal outcome [41–43], daclizumab was withdrawn from the market by the manufacturer in March 2018. The EMA recommended the immediate suspension and recall of daclizumab. Follow-up for at least 6 months after treatment with DAC-HYP was mandatory.

All cases of encephalitis were spontaneous reports, except for the three cases which came from the EXTEND trial [35], and showed significant heterogeneity. At least five of these patients had clinical symptoms compatible with DRESS syndrome with CNS involvement [41, 44], while others demonstrated evidence of CNS vasculitis or were associated with anti-N-methyl-d-aspartate (anti-NMDA) or anti-glial fbrillary acidic protein (anti-GFAP) antibodies [37, 43, 45]. One encephalitis have also been reported months after stopping treatment, suggesting that vigilance for secondary autoimmunity may need to be extended for many months after drug cessation [46].

##### Comment

World-wide market approval of DAC-HYP was based on data of short-term clinical trials (CHOICE 6 months, SELECT one year, DECIDE 2–3 years) [30–32] showing that the treatment benefits on clinical and MRI disease measures outweighed the risks. Following the long-term follow-up in EXTEND [35], additional rare but serious inflammatory syndromes were identified that were not apparent in previous pivotal trials.

The example of daclizumab illustrates quite well that there is no guarantee that a therapy which has been proven to be effective and safe for use in one condition, will work with the same safety profile in another condition. Daclizumab was initially developed as an intravenous therapy (Zenapax®) to prevent acute organ rejection in patients with de novo allogeneic renal transplantation. While Zenapax® has shown excellent tolerability and long-term safety in kidney transplant patients [47–49], this was not the case for Zinbryta™ in MS patients. One reason could be the fact that Zenapax® was only used in combination with cyclosporine and corticosteroids, thereby preventing the development of serious autoimmune and inflammatory events. Another likely reason could be differences in the immunogenetic background of both diseases [50–54]. This would mean that blocking the same IL-2R-HA α-chain likely triggers different immunogenetically predisposed cytokine-signalling pathways.

Even if the precise mechanisms of these serious inflammatory syndromes under Zinbryta™ still need to be clarified, various theories have been postulated.

Both CD56bright NK and CD4^+^CD25^+^Foxp3^+^ Tregs cells play a major role in controlling T cell responses and thereby maintaining homeostasis [55–57]. While daclizumab leads to an expansion and stimulation of CD56bright NK cells, it reduces the number of circulating CD4^+^CD25^+^Foxp3^+^ Tregs cells without affecting their function [58, 59].

On the one hand, diminished cell-surface expression of the activating receptor DNAM-1 (DNAX Accessory Molecule-1), which has been recently identified as a crucial player in NK- and Treg-cell mediated control of T cell activity, on NK [60] and Treg [61] was suggested to result in impaired immune regulatory function [40]. On the other hand, it was speculated that a predicted daclizumab-induced decline of CD4^+^CD25^+^Foxp3^+^ Treg cells without an expected concomitant expansion of immunoregulatory CD56bright NK cells (probably genetically determined) leave Zinbryta™-treated patients vulnerable to severe inflammatory syndromes [28, 38, 62].

Moreover, the story of daclizumab demonstrates the difficulty of detecting rare SAEs in clinical trials and highlights the importance of phase IV studies (post-marketing surveillance trials) of newly approved treatments [4, 46, 63, 64]. Thus, clinical trials can only provide limited exposure of an investigated drug to a highly selected and controlled cohort. Identification of rare AEs may, however, require a more heterogeneous patient group, with more potential for drug-drug and drug-other condition interactions, treated for longer periods of time before such events can be recognized [42].

Although SAEs were also observed with other DMTs, daclizumab is the first licensed MS biological agent that was permanently withdrawn after regular approval from the world-wide market [46]. Natalizumab, for example, was withdrawn in 2005 after the first three cases of PML, however reintroduced under strict monitoring requirements 1 year later. While two decades ago there was a substantial unmet need for new DMTs because treatment options for MS patients were limited, a wide range of therapies is now available. Natalizumab might not be licensed if it was introduced today, and the safety profile of daclizumab might have been evaluated differently if it had been the first highly effective MS treatment introduced to the market.

### B Cells Targeting mAbs

Evidence has accumulated, especially over the past 10–15 years, that strongly implicates the involvement of B cells in MS pathophysiology [65–68]. This view is based on neuropathological, serological, and immune cellular findings demonstrating abnormal antibody presence in CSF and lesions, CNS B cell infiltration, abnormal pro-inflammatory cytokine production by B cells, increased levels of B cell-relevant cytokines and chemokines in CSF of MS patients, and strinking results from clinical trials on B cell depletion therapies [65, 68–70]. Multiple approaches were proposed for modulating B cell populations in MS patients, including CD19^+^ (inebilizumab) and CD20^+^ mAbs (rituximab, ocrelizumab, and ofatumumab), agents targeting B cells survival factors (atacicept, tabalumab, Ianalumab, and belimumab), and inhibition of Bruton’s tyrosine kinase. In addition, all DMTs approved for MS have been found to exert suppressive or immunomodulatory effects on B cells [71].

#### Tabalumab

##### Background

Receptor engagement by B cell activating factor (BAFF), also known as tumour necrosis factor ligand superfamily member 13B (TNFS13B/CD257), leads to multiple immune cell functional activities including promotion of T cell activation, maturation and survival of B cells [72]. BAFF is the dominant homeostatic regulator of peripheral B cells [73]. It binds to three receptors, namely, the transmembrane activator, calcium modulator, and cyclophilin ligand interactor (TACI/TNFSR13C/CD267) receptor; the BAFF receptor (BAFF-R/TNFSR13B/CD268); and the B cell maturation antigen (BCMA/TNFSR17/CD269) [74, 75]. BAFF binds to the BAFF-R with significantly higher affinity than to the BCMA and TACI [75].

Tabalumab (LY2127399) is a human IgG4 mAb that neutralizes membrane-bound and soluble BAFF [76].

##### Studies

In the phase II dose-ranging study (NCT00882999) evaluating the efficacy and safety of tabalumab in patients with RRMS, participants were randomized to receive subcutaneously either one of four doses of tabalumab (4 mg (*n* = 35), 12 mg (*n* = 34), 40 mg (*n* = 34) or 120 mg (*n* = 36)) or placebo (*n* = 35) every 4 weeks for a study duration of 73 weeks. The pSE was mean total cumulative number of GELs (whether new, pre-existing, unchanged, or enlarged from previous scans), summed over weeks 12, 16, 20, and 24 (Table 1) [77]. Of the 245 included patients, 197 (80%) completed the study. The study failed to meet its pSE, as the differences overall, or between any of the tabalumab groups and placebo, were not statistically significant. Furthermore, there was no indication of any treatment effect on secondary outcomes, including development of new/enlarging T2 hyperintense lesions or the ARR. However, the proportion of patients reporting at least one treatment-emergent AE, SAE, and follow-up emergent AE was higher in tabalumab treated patients compared to placebo (results currently not further specified) [77]. Full publication is still pending.

The anti-BAFF-R fully human IgG1 mAb lanalumab (VAY736) was recently tested in a phase II study (NCT02038049) assessing the efficacy of a single infusion of lanalumab on disease activity as measured by MRI in RRMS (Table 1). In this study, patients were randomized to receive an infusion of VAY736 (10 mg/kg) or placebo. The pSE was the cumulative number of new GELs on brain MRI scans at weeks 8, 12, and 16. However, the study recruitment was terminated based on strategic considerations after eight patients were enrolled (originally recruitment of 96 patients was planned). The results are still pending.

##### Comment

The clinical study on tabalumab illustrates the complex nature of B cell actions and effects on the autoimmune system [69] and highlights the importance of memory B cells (Bmem) as central players in the MS immunopathogenesis [73, 78, 79]. Bmem display diverse effector functions including cytokine production, antigen presentation, and serving as antigen-experienced precursors to antibody-secreting cells [78].

Blockade of BAFF by tabalumab induced depletion of naïve/mature B cells and a dose-dependent increase in Bmem [73]. Therefore, the missing treatment effect of tabalumab in RRMS may be explained by the fact that compared with other B cell targets such as CD20^+^ cells, Bmem are spared under treatment with tabalumab, and T cell activation may be preserved [73].

Given the importance of BAFF-R signalling pathways in B-cell survival and homeostasis, the anti-BAFF mAb belimumab, which is already approved for the treatment of systemic lupus erythematosus and active lupus nephritis, is actually tested in a phase II study (NCT04767698) in RRMS patients (Table 1). Forty patients will be randomized 1:1 to either receiving ocrelizumab (300 mg two infusions 2 weeks apart at baseline and then 600 mg as a single infusion every 6 months) or belimumab (200 mg subcutaneous weekly for 36 months) plus two courses of ocrelizumab (300 mg two infusions 2 weeks apart at baseline and 600 mg as a single infusion 6 months later). The investigators hypothesize that belimumab, given in addition to a short course of ocrelizumab will be safe and less immunosuppressant as measured by antibody response to pneumococcal vaccination as compared to continuous treatment with ocrelizumab.

### Cytokine-Directed Therapies

Preclinical and clinical data provide evidence that cytokines play a major role in the pathogenesis of MS [80, 81]. However, the disease is not governed by any one particular cytokine but instead involves a complex interplay between pro- and anti-inflammatory cytokines [80, 82, 83]. Therefore, multiple attempts have been made to treat MS patients with recombinant anti-inflammatory cytokines, or inhibitors of pro-inflammatory cytokines [80]. To date, only interferons have been approved as cytokine-directed therapy for MS.

#### Secukinumab (AIN457)

##### Background

Based on evidence from human and animal studies, there is a substantial body of literature suggesting a key role of the pro-inflammatory cytokine Interleukin (IL)-17 in MS pathophysiology [84–88].

IL-17A is the key effector cytokine produced by pro-inflammatory T helper 17 (Th17) cells, CD8^+^ and γδ T cells and by other cells in the CNS, such as astrocytes and oligodendrocytes in active MS lesions [89]. IL-17A drives expression of several inflammatory genes and mediator release, and adversely affect function in different cell types in the CNS such as microglia, astrocytes, oligodendrocytes, neurons, neural precursor cells, and endothelial cells [90]. Thus, IL-17A stimulates different cell types including fibroblasts, endothelial and epithelial cells to produce pro-inflammatory mediators which lead to an overall leukocyte activation, tissue inflammation, and destruction [87, 88, 91]. Moreover, it can recruit and activate neutrophils, microglia, and astrocytes to produce pro-inflammatory cytokines and chemokines [92]. IL-17A blocks proliferation of neural precursor cells resulting in a decrease of astrocytes and oligodendrocyte precursor cells (OPCs), thereby hindering remyelination and neural repair in the CNS [93].

Increased proportion of Th17 cells, as well as increased levels of IL-17A (protein and messenger RNA), were observed in the brain tissue of MS patients, especially in acute and chronic active lesions, compared to healthy controls [89]. The concentration of IL-17 and Th17 cells is significantly increased in the peripheral blood and CSF of MS patients, especially during relapses, but also in remissions, compared to healthy subjects [85, 94–96] and correlates with clinical and paraclinical disease activity [85, 97].

All current MS DMTs hamper Th17 cells, however, to different degrees regarding maturation stages and phenotypes [85]. Secukinumab or AIN457 is a fully human IgG1κ mAb that binds to human IL-17A, neutralizing its bioactivity. Secukinumab is already approved for treatment of psoriasis, psoriatic arthritis, and ankylosing spondylitis.

##### Studies

In the phase II study (NCT01051817) evaluating the efficacy and safety of secukinumab in patients with active RRMS (one GEL at screening/baseline MRI, one relapse in the last year, or two relapses in the last 2 years) over 6 months, participants were randomized to either receive 10 mg/kg (*n* = 38) or placebo (*n* = 35) intravenously administered at weeks 0, 2, 4, 8, 12, 16, and 20 [98]. The pSE was the cumulative number of combined unique active lesions (CUAL) defined as new GELs and new/enlarging T2-weighted lesions without double counting, observed on monthly brain MRI from week 4 to week 24 (Table 1). 61 participants completed the study (35 of the secukinumab and 26 of the placebo group). The trial failed to reach its pSE. However, secukinumab reduced significantly the mean cumulative number of new GELs from week 4 to week 24 and at each monthly visit starting at week 16 through end-of-study visit (= week 36) and the mean cumulative number of all GELs at weeks 12, 16, 20, 24, and 36 compared to placebo. Secukinumab also reduced the mean cumulative number of new/enlarging T2-weighted lesions compared to placebo, with a significant difference between treatments observed at end-of-study visit (but not beforehand) [98]. The ARR was reduced in secukinumab compared to placebo; however, the difference was not significant in this trial which was not powered to assess relapse rates. The rate of AEs was comparable to placebo, although mild-to-moderate infections were more frequent in the secukinumab group. No SAEs were reported [98].

After completing the core study, patients received secukinumab 10/mg/kg intravenously every 4 weeks in the 12-month open-label extension study (NCT01433250). The pSE was number of subjects with AEs and the number of abnormalities in safety assessments (Table 1). However, further enrolment was stopped after inclusion of 39 patients. The sponsor indicated that this decision was not related to safety or tolerability concerns observed in the study. Unfortunately, the results of the extension study are not fully published.

Based on the results of the previous core study, the efficacy of secukinumab was assessed in another adaptive dose-ranging 6-months phase II study (NCT01874340) in patients with active relapsing multiple sclerosis (RMS). In stage 1, patients were randomized 1:1:1:1 to infusion of secukinumab (3, 7, or 15 mg/kg) or placebo. Secukinumab was given on days 1 and 15, week 4, and every 4 weeks thereafter. In stage 2, patients should be randomized 1:1:1 to two secukinumab doses (either 7 and 3 mg/kg, or 15 and 7 mg/kg) selected after an interim analysis during stage 1, or placebo [99]. However, the trial was terminated early after enrolment of only 28 patients, and effects on outcome measures could not be determined (Table 1). The sponsor indicated that this decision was based on the development of another anti-IL17 mAb, called CJM112, with superior potential (50–100-fold higher in vitro affinity to IL-17A) to secukinumab. The 6-month phase II proof-of-concept study (MABINGO) intended to evaluate the efficacy of CJM112 relative to fingolimod in controlling brain MRI disease activity in RRMS who discontinued their natalizumab therapy for any reason other than lack of efficacy or presence of neutralizing antibodies against natalizumab [100]. Patients would have been randomized 1:1:1 to high or low dose of CJM112 (subcutaneous application on day 1 and 15, month 1 and then monthly) or fingolimod (0.5 mg oral, once daily) [100]. The pSE was the cumulative number of new GELs on brain MRI scans at months 4, 5, and 6 after start of investigational treatment (Table 1). However, the trial was not executed due to a company policy decision.

##### Comment

The efficacy of secukinumab on MRI data in the small core study (NCT01051817) is particularly difficult to judge due to imbalances between both groups at baseline. Thus, the secukinumab group included a subcohort with very high MRI disease activity at baseline, because the mean number of GELs was higher in the secukinumab than in the placebo group, while at the same time, a lower percentage of patients of the secukinumab group had GELs. Taking into account that the mean number of T2 lesions at baseline and mean number of relapses in the previous 2 years were largely comparable between both groups, the MRI treatment effects in this trial were particularly driven by the subgroup of patients highly active at baseline, leading to an overestimation of the therapeutic efficacy [101]. Moreover, the secukinumab group consisted of more male and older participants compared to the placebo group. This could be associated with an inferior response to MS therapy and might therefore have partially masked the treatment effect of secukinumab [101].

However, analyzing the cumulative number of new and all GELs over time and restricting analyses of CUAL and GELs to MRIs obtained from weeks 12 to 24, the study demonstrated clear evidence for a ‘delayed’ therapeutic effect, staying pronounced from week 12 [101].

Development of novel cytokine-directed therapies remains complicated as cytokine networks are complex and individual cytokines may have diverse and even opposing functions in different clinical scenarios [81]. The MABINGO study would have clarified what relevance IL-17 or Th17 cells really play in MS. However, this question still remains outstanding due to the unfortunate company policy decision to halt the CJM112 program. Recently, we were able to demonstrate that long-term natalizumab treatment (i.e., more than one year) is associated with an increased pathogenicity of Th17 cells, which might explain the clinical phenomenon of disease rebound upon natalizumab treatment cessation [102]. In this context, the CJM112 trial might have been an important step towards a more personalized selective treatment approach in MS, i.e., identification of those patients whose MS disease is especially IL-17 driven and initiation of a specific anti-IL-17 therapy with CJM112.

### Antiviral approaches

Strong evidence suggests that MS may be triggered by microbial infections [103]. Pathogens associated with development or exacerbation of MS include bacteria, such as Chlamydia pneumonia and Staphylococcus aureus-produced enterotoxins, myxoviruses, herpes viruses (Epstein-Barr virus, human herpes virus 6, varicella–zoster virus, cytomegalovirus), and human endogenous retroviruses (HERVs) [104]. While until recently no single pathogen has been accepted as causal agent for MS [105], a latest study on more than 10 million young adults could identify Epstein-Barr virus as one of the leading trigger of MS [106].

#### Temelimab (GnbAC1)

##### Background

About 8% of the human genome is composed of DNA sequences of HERVs that have been acquired along the last 100 million of years through multiple integrations by now extinct exogenous retroviruses [107]. Under physiological conditions, these elements are frequently inactive or non-functional due to deactivating mutations and epigenetic control. However, if uncontrolled and/or abnormally activated, HERVs can lead to detrimental effects in their host organisms by altering gene expression profiles, expressing pathogenic nucleic acids/proteins or even inducing deleterious mutations. Once of these proteins, the pathogenic HERV Type-W envelope protein (pHERV-W ENV), is known to drive immune activation, inflammation, and neurodegeneration in MS, and to inhibit remyelination [108, 109]. Interestingly, increased concentrations of the pathogenic HERV Type-W envelope protein (pHERV-W ENV) can be found in peripheral blood, CSF, and cerebral lesions of MS patients [110].

Temelimab or GNbAC1 is a recombinant humanized IgG4 mAb against the pHERV-W ENV. Preclinical and phase I/IIa studies of temelimab indicated good tolerability without signs of immunogenicity, accompanied by the protection of oligodendroglial precursors and decreased levels of proinflammatory cytokine production [111–115].

##### Studies

In the phase IIb trial CHANGE-MS (NCT02782858), 270 RRMS patients were randomized equally to receive either one of three doses of temelimab (6, 12, or 18 mg/kg) or placebo intravenously every month for 24 weeks (period 1); at week 24, placebo-treated participants were re-randomized to treatment groups (period 2). This dosing scheme was continued during the subsequent 48-week extension study ANGEL-MS (NCT03239860) [116] (Table 1).

The trial failed to reach its pSE: the cumulative number of GELs in brain MRI scans identified from weeks 12 to 24 did not significantly differ between the three temelimab doses and placebo. Only in participants showing GELs at baseline, a trend towards a reduction in new GELs was reported for the highest dose of temelimab (18 mg/kg) compared to placebo at one time point in the 24-week follow-up. Moreover, treatment groups did not significantly differ in the number of new/enlarging T2 hyperintense lesions in the periods 1 and 2 and in the extension study.

At the end of the study, participants treated with 18 mg/kg temelimab had fewer new black holes and showed statistically significant reductions in cortical and thalamic atrophy, and decrease of myelin integrity, as measured by magnetization transfer ratio, compared with the placebo/comparator group (all participants initially randomized to placebo and re-randomized to any dose of temelimab in period 2). Patients of the different treatment groups did not significantly differ from the placebo group regarding ARR, disability progression/improvement, or proportion of reaching ‘no evidence of disease activity’ (NEDA) [116].

##### Comment

Temelimab does not seem to be able to treat the inflammatory-driven part of MS (represented by relapses, GELs, T2 lesions). However, the results of continued improvements in the MRI-based neurodegenerative outcomes, such as brain volumes, magnetization transfer ratio, and black holes in CHANGE- and ANGEL-MS, suggest a possible, dose-dependent neuroprotective effect of temelimab at the highest dose of 18 mg/kg compared with the placebo/comparator group. The fact that the subgroup of inactive participants (no GELs at baseline) treated with 18 mg/kg temelimab showed a similar relative reduction in whole and regional brain atrophy as the overall study group could be interpreted as supportive evidence of a neuroprotective capacity independent of an effect on inflammation. In this context, a new phase IIa study (ProTEct-MS, NCT04480307) will be conducted in RMS patients who received rituximab for at least 12 months but still experience progression in absence of relapse activity (PIRA) (Table 1). RMS patients will be randomize equally to receive either one of three doses of temelimab (18, 36, or 54 mg/kg) or placebo intravenously every months for 48 weeks.

Despite the possible neuroprotective effect of temelimab, it is important to bear in mind that previous studies (CHANGE- and ANGEL-MS) do not definitively answer if pHERV-W is a leading trigger for MS development.

### Blocking Lymphocyte Trafficking

#### Vatelizumab

##### Background

Integrins are the foremost family of cell adhesion and signalling proteins that regulate immune cell trafficking in humans [117]. The α2β1 integrin, also known as GPIa/IIa, CD49b, or very late antigen (VLA)-2, is a collagen-binding molecule expressed on numerous different cell types, including epithelial cells, endothelial cells, fibroblasts, and hematopoietic elements, including platelets and specific subsets of leukocytes.

Vatelizumab is a mAb directed against the α2 subunit of VLA-2 on activated, not resting lymphocytes. The exact mechanism of action of vatelizumab is not known, but it is assumed that vatelizumab blocks the interaction of VLA-2 expressed on activated lymphocytes with collagen fibres that build up in inflammatory sites [118]. Thereby, it prevents the formation of inflammatory lesions without affecting immune-cell migration into the CNS, and the triggering of an inflammatory cascade including cytokine release and recruitment of additional inflammatory cells at sites of inflammation [118]. Moreover, vatelizumab was demonstrated to induce CD4^+^FoxP3^+^ Treg cells [119].

##### Studies

In the phase IIa/IIb trial EMPIRE (NCT02222948 and NCT02306811), 112 RRMS patients were randomized 1:1:1:1:1 to receive placebo or one of four vatelizumab doses (400, 800, 1200, or 1600 mg) intravenously at baseline and at weeks 2, 4 and 8, and then every 4 weeks during the extension study. The treatment period was 12 weeks, followed by an optional long-term extension study or alternatively safety follow-up period of up to 92 weeks. The pSE was the reduction in the cumulative number of new GELs on MRI from week 4 to week 12 in each dose arm, compared with placebo (Table 1). Secondary endpoints include the evaluation of the safety, tolerability, and pharmacokinetics of vatelizumab compared with placebo [120]. The study was discontinued based on planned interim analysis of the pSE, and not due to safety concerns (Table 1). Results of the study are unfortunately not published.

##### Comment

Based on the successful clinical experiences with natalizumab, the migration process of immune cells into the inflamed CNS tissue has been brought to the fore of pharmacological research [22]. Natalizumab already provided evidence of a strong efficacy in treating RRMS [121–128]. The idea behind the development of vatelizumab was to find a more ‘clever’ agent that is simultaneously as efficacious as natalizumab, but without the side effect of PML.

Because the circumstances leading to the discontinuation of the study have not been made public, we can only speculate about the reasons. Perhaps, EMPIRE was underpowered because the study was weakened by using four therapeutic doses with sample sizes of 22 patients per treatment arm. In animal model, it was shown that VLA-2 blockade was only effective when administered in the early symptomatic stage of experimental allergic encephalomyelitis (EAE), while being rather ineffective when given either too early or too late in the course of the disease [129]. Perhaps, the optimal time for anti-VLA-2 administration to prevent the formation of inflammatory lesions in the CNS was missed in EMPIRE. Against the background that vatelizumab may not penetrate into the CNS due to the blood–brain-barrier (BBB), which is only disrupted in already inflamed areas [130], vatelizumab perhaps does not exert its function in the too early or very late stages of MS.

### Neuroregenerative Approaches

#### Opicinumab

##### Background

The leucine-rich repeat and immunoglobulin-like domain-containing Nogo receptor-interacting protein-1 (LINGO-1) is a membrane protein, expressed selectively in the CNS on oligodendrocytes, with higher levels found in OPCs and neurons. LINGO-1 expression is upregulated in various animal models with CNS injury and in human CNS diseases [131]. Across diverse animal CNS disease models, inhibition of LINGO-1 facilitates oligodendrocyte differentiation and survival, and is associated with axonal regeneration, neuronal survival and regeneration, remyelination, and functional recovery [131–135]. Based on these promising preclinical studies, the human IgG anti-LINGO-1 mAb opicinumab (also known as BIIB033) was trialled in several clinical studies in both optic neuritis and MS.

Two phase I studies, a single ascending–dose study in healthy volunteers and a multiple-dose study in patients with RRMS or SPMS demonstrated a tolerable safety profile [136] and led to the development of three phase II trials.

##### Studies

In the phase II study RENEW (NCT01721161), patients with first unilateral acute optic neuritis (AON) were randomised 1:1 to receive either 100 mg/kg opicinumab (*n* = 33) or placebo (*n* = 36) once every 4 weeks up to week 20 following standard high-dose treatment with intravenous methylprednisolone. The pSE was the remyelination at 24 weeks, measured as recovery of affected optic nerve conduction latency using full-field visual evoked potential (FF-VEP) versus the unaffected fellow eye at baseline (Table 1) [137]. Even if the study failed to reach its pSE, opicinumab treatment significantly ameliorated P100 latency at week 32 compared to placebo [137].

In the phase II trial SYNERGY (NCT01864148), 330 RRMS and 89 SPMS patients with relapses were randomly assigned in a 1:2:2:2:2 ratio to either 3 mg/kg (*n* = 45), 10 mg/kg (*n* = 95), 30 mg/kg (*n* = 94), or 100 mg/kg (*n* = 92) opicinumab or placebo (*n* = 93) once every 4 weeks. All participants received concurrent treatment with intramuscular IFNβ-1a once weekly. In this study, opicinumab missed the pSE, the percentage of participants achieving the confirmed disability improvement (CDI), which was a multicomponent endpoint measured by Expanded Disability Status Scale (EDSS) [138], the Timed 25-Foot Walk (T25FW) [139], the Nine-Hole Peg Test (9HPT) [140], and the 3 s Paced Auditory Serial Addition Test (PASAT-3) [141], over the 72-week treatment period (Table 1) [142]. Compared with placebo, CDI did not differ with 3 mg/kg or 100 mg/kg opicinumab, with weak evidence seen with 10 mg/kg opicinumab and some improvement with 30 mg/kg opicinumab. A significant dose-linear improvement could not be observed. Evaluation of the secondary endpoint (percentage of patients with confirmed disease worsening measured by the same tests) demonstrated no effect of opicinumab over 72 weeks. Using an overall response score, an integrated assessment of CDI and CDP based on EDSS, T25FW, 9HPT, and PASAT-3 as tertiary endpoint, improvements were seen at week 24 and 36, with all doses of opicinumab, with the largest effect under 10 mg/kg, compared to placebo.

In the 72-week phase II trial AFFINITY (NCT03222973), 263 RRMS and SPMS patients were randomized to intravenous 750 mg opicinumab, corresponding to a dose of 10 mg/kg, every 4 weeks, or placebo as an add-on to IFNβ, dimethyl fumarate, or natalizumab. The pSE was the overall response score, an integrated assessment of CDI and CDP based on EDSS, T25FW, and 9HPT (Table 1). In October 2020, Biogen announced that the AFFINITY trial failed to meet its primary and secondary endpoints and discontinued development of opicinumab [143].

##### Comment

While animal models provide the experimental flexbility to analyse mechanism of remyelination, measuring the effect of remyelination-promoting approaches in humans is more challenging [144, 145]. Even if the ‘proof of concept’ was demonstrated by the positive results of opicinumab at week 32 in RENEW, this example raises the following questions as to the therapeutic goal of remyelination [145].

The first question is to what extent does this mAb, which has shown robust remyelination in demyelination animal models [131, 133], really reach the CNS? It seems as if opicinumab given at higher doses crosses the BBB, at least to a certain extent, as seen by the positive results at week 32 in RENEW [137].

Another question that arises in this context is what the ideal readout for ‘proof of concept’ is. Measuring the effect of remyelination-promoting therapies by measuring the P100 latency in a white matter tract as the optic nerve by using FF-VEP is more promising than measuring remyelination in MS lesions or certain areas of the brain by using advanced imaging techniques, or indirectly by measuring changes of disability scores which especially depend on gray matter damage [146–148]. Moreover, advanced imaging techniques such as magnetisation transfer ratio and diffusion tensor imaging [142] are both somewhat non-specific and can be influenced by changes in water content, oedema, and inflammation [149].

What is the appropriate point in time to initiate a remyelination-promoting therapy? Several studies suggest that remyelination starts immediately after immune damage and is finished within several weeks or months [150]. Consistent with this hypothesis, a subgroup analysis of the RENEW trial demonstrated better outcomes in patients who received the first dose of opicinumab shortly after AON onset (< 25 days) [151]. Therefore, it can be speculated that remyelinating treatment should start as soon as possible after a demyelinating event, and thus at onset of first symptoms to prevent long-term axonal degeneration [145, 150].

Moreover, there is a lack of experience concerning the appropriate selection of patients who might profit from remyelination-promoting approaches. In the RENEW study, opicinumab was more efficient in older AON patients (aged ≥ 33 years) and those with more severe visual impairment at baseline [151]. The weaker intrinsic remyelination in older participants could explain the greater therapeutic effect of opicinumab in this subgroup [151]. In contrast, in the SYNERGY study, participants at a younger age with shorter disease duration (≤ 20 years since symptom onset) and clinical and MRI features suggestive of more preserved brain tissue responded better to opicinumab [142]. The efficiency of myelin repair by oligodendrocytes declines with age due to impaired differentiation of OPCs into sheath-forming oligodendrocytes or impaired recruitment of these cells to demyelinated axons at the lesion site. Therefore, opicinumab potentially restores differentiation of still available OPCs that might become insufficient more frequently in older patients. Shorter disease duration and more preserved brain structure are in line with reports defining more intact CNS axons as a prerequisite for myelin repair [150, 151].

Is remyelination really a clinically relevant therapeutic strategy? Remyelination-promoting approaches will only help given in addition to DMTs and very early in the course of MS before any neurodegeneration or gray matter damage occur. There is, however, increasing evidence that gray matter damage can already occur early in the course of disease [152]. While white matter as the optic tract in the RENEW trial is able to regain full functionality, gray matter lesions and damage of associated complex neuronal networks are very likely not to be fully restituted by remyelination [153].

## Failed mAbs in Progressive Multiple Sclerosis with Otherwise Approved and Working Compounds

### Natalizumab

#### Background

To migrate across the BBB into the CNS, autoreactive immune cells need to express certain surface proteins (α4β1-integrin) that allow binding to the vascular cell adhesion molecule 1 (VCAM1), which is expressed on the inflamed endothelium of cerebral blood vessels. Natalizumab is a recombinant humanized mAb directed against α4-integrin (VLA-4) on leukocytes [154] blocking adherence to the lumen of the endothelial BBB and transmigration of B and T lymphocytes, macrophages, and dendritic cells into the CNS, and decreasing cerebral microglial activation [155, 156]. This mechanism may reduce chronic intrathecal (meningeal and CNS parenchymal) inflammation and presented the rationale to test the efficacy of natalizumab in SPMS [155]. Early Ib/IIa studies in SPMS and PPMS indicated reduced cortical microglial activation and biomarker of intrathecal inflammation, axonal damage, and demyelination, and improved disability (assessed by EDSS) and ambulation (assessed by T25FW) following natalizumab [155, 157–159].

#### Studies

The phase IIIb ASCEND trial (NCT01416181), with an optional 2-year open-label extension, aimed to assess whether natalizumab slows disease progression in SPMS, independent of relapses [160]. Enrolled patients had SPMS for a minimum of 2 years and showed disability progression unrelated to relapses in the year before study inclusion. 889 participants were randomly (1:1) assigned to receive intravenous natalizumab (*n* = 440) or placebo (*n* = 449) every 4 weeks for two years (part 1). Patients completing part 1 could enrol in part 2 (continuing natalizumab, *n* = 291; initiating natalizumab, *n* = 274), in which all patients received natalizumab every 4 weeks until the end of the study. The pSE in part 1 was the multicomponent measure of CDP over the 96-week treatment period comprising the EDSS, T25FW, and 9HPT, which were done every 12 weeks. The primary outcome in part 2 was the incidence of AEs and SAEs (Table 1) [160].

Natalizumab showed no treatment effect on the multicomponent CDP, nor on its EDSS or 25TWT components, but reduced the disability progression of the upper limb component assessed by the 9HPT. Significant reductions in ARR and MRI measures of focal inflammation were also documented. Natalizumab did not affect whole brain atrophy during part 1, but significantly reduced whole atrophy when considering parts 1 and 2 together or part 2 separately. The incidence of AEs and SAEs did not differ between natalizumab and placebo in part 1 and between patients continuing natalizumab and those initiating natalizumab in part 2 [160].

#### Comment

Natalizumab is a highly efficacious therapy for the treatment of RRMS, as demonstrated in clinical trials and numerous real-world studies [121–128]. However, natalizumab failed to demonstrate a significant effect on disability progression in SPMS in contrast to siponimod in the EXPAND trial [161]. Study populations of the ASCEND [160] and EXPAND trial [161] were quite comparable (similar mean age, disease duration, EDSS, sex distribution, and proportion of patients with GELs in baseline brain MRI) with a higher proportion of more disabled patients with EDSS scores of 6.0–6.5 (63% vs 56%), and longer period of time since conversion to SPMS (4.9 vs 3.9 years) in ASCEND compared to EXPAND. Despite such shared characteristics and their common reducing effect on clinical and radiological signs of focal inflammation (ARR and the occurrence of GELs and new/enlarging T2 lesions), only siponimod significantly reduced the risk of disability progression. The example of natalizumab illustrates very clearly that the pathophysiological mechanisms of PIRA are independent of leukocyte ‘outside-in’ traffic while this feature is the most relevant component for driving relapses and relapse-associated worsening (RAW). The dominant feature of the pathology of progressive MS is the presence of a compartmentalized inflammation [162–164]. Because siponimod can cross the BBB in contrast to natalizumab, it can strongerly influence the inflammation on site in the CNS.

In contrast to several previous trials in SPMS [165–169], the ASCEND trial used a multicomponent study endpoint which is more sensitive and possibly specific to changes in disability than the EDSS alone [170–172]. The failed treatment effect on EDSS is not surprising because the EDSS has been shown to have poor responsiveness to disease progression in patients with higher baseline EDSS scores [173]. Because most patients of the ASCEND trial already suffered from a strongly limited walking ability (63% of study subjects had an EDSS either 6.0 or 6.5), the 25TWT also showed little sensitivity as it might have ceiling effects for those patients [139]. As predicted by the length-dependent axonopathy hypothesis [172], natalizumab had no effect on lower limb function (EDSS and T25FW), but a statistically significant treatment effect on upper-limb disability progression as measured by the 9HPT [172]. This hypothesis indicates that neuronal domains with longer central axonal projections are more likely to be involved early in the clinically apparent progressive phase of MS [172]. Therefore, future trials on progressive MS might need to consider longer treatment phases to capture the potential benefits in all key aspects of disability and to implement domain­specific outcome measures that shift the focus to shorter tract-based pathways as upper limb, cognitive, and visual outcomes.

### Rituximab

#### Background

Rituximab is a mouse-human chimeric mAb directed against the CD20 antigen expressed on the surface of most B cells except B-cell progenitors (pro-B cells) and differentiated plasma cells [174]. It depletes CD20^+^ B cells through a combination of ADCC, antibody-dependent cellular phagocytosis (ADCP), complement-dependent cytotoxicity (CDC), and induction of cell apoptosis [174]. Rituximab was the first anti-CD20 mAb developed for use in humans in 1997 and received FDA approval for B-cell non-Hodgkin lymphoma, chronic lymphocytic leukemia, rheumatoid arthritis, granulomatosis with polyangiitis, microscopic polyangiitis, and pemphigus vulgaris, but not for MS.

#### Studies

In the 48-week phase II trial HERMES (NCT00097188), a single course of intravenous rituximab (1000 mg on days 1 and 15, *n* = 69) was shown to significantly reduce the number of total (= pSE) and new GELs, the T2 lesion volume, the proportion of patients with relapses at week 24 and 48, and the ARR from baseline to week 24, but not from baseline to week 48 in RRMS patients compared to placebo (*n* = 35) [175]. In the phase II/III study OLYMPUS (NCT00087529), 439 PPMS patients were randomized 2:1 to receive two intravenous infusions of rituximab 1 g each, 2 weeks apart (*n* = 292), or placebo (*n* = 147) every 24 weeks through 96 weeks [176]. The trial failed to meet its pSE since there was no significant reduction in time to 12-week CDP after 96 weeks (Table 1). Although one secondary MRI endpoint, reduction in T2 lesion volume, was reached, the other, brain volume change, was not different from placebo [176]. Compared with placebo, rituximab patients had less worsening in the multiple sclerosis functional composite (MSFC) scale [177], T25FW, 9HPT, and PASAT. A subgroup analysis indicated a statistically significant effect of rituximab on time to CDP and on T2 lesion volume in patients younger than 51 years and/or in those with GELs at baseline (Table 1). Moreover, patients with a short disease duration (≤ 3 years) receiving rituximab had a longer time to CDP compared to patients receiving placebo [176]. Despite the promising results in PPMS, as well as in RRMS, the clinical development of rituximab was interrupted. However, in the light of the well-established long-term safety profile of rituximab from its wide use in other diseases and of the promising results obtained in MS, researchers were highly motivated to pursue further clinical trials.

In the small single-centre phase II study, add-on therapy with rituximab (375 mg/m^2^ weekly × 4 doses) induced a significant reduction of ARR and GELs and improvement of MSFC and PASAT in 32 RRMS patients with breakthrough while receiving IFNβ-1a/b or glatiramer acetate [178]. In the open-label, phase II trial STRIX-MS (EudraCT 2010–023,012-38), 75 patients with clinically stable RRMS (no evidence of relapse or worsening) under treatment with IFNβ or glatiramer acetate for at least 6 months were switched to rituximab (two doses 1000 mg 2 weeks apart) and followed by repeated clinical assessments, MRIs, and measurement of neurofilament light chain (NFL) concentrations in CSF for 24 months [179]. Rituximab was shown to have an equal or superior effect in reducing inflammatory activity in RRMS measured by MRI and CSF-NFL compared to first-line injectables during the first year after treatment shift and to induce improvement in treatment satisfaction [179]. In the phase I/II trial RIVITaLISe (NCT01212094), 27 SPMS patients without relapses in the preceding year and nonremitting/sustained progression of disability over 3 months were assigned to receive either combined intravenous and intrathecal rituximab (*n* = 18) or placebo (*n* = 9) [180]. Intrathecal rituximab was applied in three single doses of 25 mg each at baseline, 1.5 months, and 12 months. The intravenous induction dose was twice 200 mg, with a 15-day interval. Of 18 patients assigned to the treatment group, 14 received at least two doses of intrathecal rituximab and were included in the interim analysis [180]. Combined intravenous and intrathecal administration of rituximab led to an almost complete and lasting depletion of peripheral B cells, while the depletion of B cells in CSF and CNS tissue was insufficiently [180]. Due to this missing efficacy, the study was stopped prematurely (Table 1). The authors hypothesized that the study might have been underpowered to detect treatment effects, especially regarding the biomarkers investigated [180]. The small phase I trial (NCT02253264) assessing the safety of intrathecal rituximab (two doses of 25 mg each at baseline and two weeks later) in 23 participants with progressive MS reached its primary safety outcome. Intrathecal rituximab resulted in a significant and sustained reduction in circulating B cells, but only a transient drop in CSF B cells and unchanged brain imaging outcomes during the 24-week follow-up period [181]. In the open-label phase II trial EFFRITE (NCT02545959), 10 patients were randomized into control group (*n* = 2), intrathecal rituximab group (20 mg) (*n* = 4), or intravenous (375 mg/m^2^) plus intrathecal (20 mg) rituximab group (*n* = 4) [182]. Patients received the therapy once and clinical, blood, MRI, and CSF assessments at several points in time during the one-year follow-up. The pSE was the change in levels of CSF biomarkers of inflammation (osteopontin) (Table 1). Secondary outcomes were changes in levels of CSF biomarkers of axonal loss (NFL), clinical, and MRI changes. The trial failed to reach its pSE because the osteopontin level remained stable in CSF; CSF level of NFL decreased only slightly at one point in time. Clinical parameters remained stable and leptomeningeal enhancements remained unchanged. Soluble CD21 (sCD21), which is a marker of B-cell pool, was decreased in serum but not in CSF after rituximab [182].

In the single-centre phase II trial GATEWAYII (NCT01569451), 55 patients with RRMS and clinically isolated syndrome (CIS) were randomly assigned to either a single cycle of rituximab (two infusions of 1000 mg 2 weeks apart) or placebo as first-line treatment, followed by subcutaneous glatiramer acetate 20 mg/daily 2 weeks later up to a maximum of 144 weeks [183]. Induction therapy with rituximab followed by glatiramer acetate was more efficient in reaching NEDA, avoiding treatment failure defined as ≥ 2 new lesions, relapses, and/or sustained accumulation of disability, and reducing MRI activity than therapy with glatiramer acetate alone [183]. Another phase II/III trial (NCT03315923), in which 84 patients with SPMS and ARR ≥ 1 were randomly assigned to receive rituximab (1000 mg every 6 months; *n* = 37) or glatiramer acetate (40 mg subcutaneous 3 times/week; *n* = 40) for 12 months, documented an apparent lack of efficacy of both treatments in controlling EDSS progression (Table 1) [184]. Both treatments could decrease the ARR and the number of active lesions in brain and cervical spine, without significant difference between both groups [184]. However, it has to be considered that the study had a short duration, and that patients randomized to glatiramer acetate had a longer disease and were older compared with those assigned to rituximab group.

#### Comment

The reason why commercialization of rituximab for MS has been abandoned by its sponsor was not due to the missed pSE in OLYMPUS, but rather due to the development of the next-generation anti-CD20 mAbs which are assumed to be less immunogenetic [185, 186]. OLYMPUS was possible underpowered to detect effects in pSE, but showed promising results in some secondary outcome measures. The experiences, which were gained with rituximab in PPMS (OLYMPUS) [176], could be used for the development of the study design of the phase III study of ocrelizumab in PPMS (ORATORIO) the same company [70]. Thus, ORATORIO included more and younger patients, had a longer follow-up, and used different statistical methods than OLYMPUS [70, 176, 187, 188]. Moreover, with respect to rituximab-treated patients, ocrelizumab-treated patients in ORATORIO had a shorter disease duration, a higher brain volume at baseline, and were treatment naive at randomization. A higher proportion of patients had GELs at baseline in the ocrelizumab group compared with the rituximab group [70, 176, 187, 188].

As other mAbs, rituximab does not pass readily across the BBB, and its CSF concentration has been estimated to reach only 0.1% of that in serum after intravenous administration [189]. Under the assumption that the compartmentalization of inflammation behind a relatively intact BBB in progressive MS prevents access through current DMTs [190], intrathecal administration of rituximab was evaluated in several clinical trials in patients with progressive MS [180–182]. The low efficacy of intrathecal administration of rituximab in previous trials might be attributed to low bioavailability (CSF rituximab levels of 2% of serum values) [180]. Whether the intrathecal dose chosen was too low or if complex mechanisms like internalization of the antibody-CD20 complex also play a role in the reduced ability of rituximab to deplete B cells remains elusive [191]. Insufficient complement and effector cells for CDD and ADCC might further explain the poor CNS efficacy of rituximab in progressive MS [180, 182, 186]. Moreover, a significant efflux of intrathecally administered rituximab into systemic circulation and an inadequate dispersion of rituximab in CSF were observed [180, 182].

The example of rituximab illustrates very well that its failure in clinical trials on progressive MS does not necessarily mean the failure of rituximab in general. An increasing body of evidence from clinical trials and real-world data suggest that rituximab is a highly effective DMT in RRMS, with a low discontinuation rate, related to a good benefit/risk profile, and a good compliance [187, 188]. Thus, rituximab as ‘first choice’ and ‘switching’ treatment offer moderate-to-large benefit against a range of other medicines in preventing relapses in RRMS [192].

Even if rituximab did not reached approval for treatment of MS, its off-label use is widespread in several countries, has dramatically increased over time, and is expected to continue to rise with the recent FDA approval of the rituximab biosimilar and its lower price compared to the reference product and other approved DMT [187, 188, 193]. It is the most commonly used DMT for all MS subtypes in Sweden [187, 194, 195] and is currently used in many low- to middle-income countries that have major barriers for accessing approved immunotherapies for MS [192]. Numerous clinical trials on rituximab are ongoing (NCT03500328, NCT03535298, NCT04047628, NCT04578639, EUCTR2020-002,981–15-DK, EUCTR2017-000,426–35-AT, NCT05078177, NCT03979456, NCT04121403, NCT04688788, NCT04578639) or completed but results are not published yet (NCT03315923, NCT02746744, NCT02545959, NCT00097188, NCT02253264). However, it is also important to note that numerous observational studies on rituximab are of poor quality [196] and the evidence about the effect of rituximab on disability worsening in all forms of MS, well-being, and serious harmful effects is very uncertain [192]. Therefore, large, methodologically rigorous studies, including randomized controlled trials, head-to-head studies, and long-term safety assessments, are needed to provide accurate safety and efficacy information of rituximab use in MS and establish global standards of care [196, 197]. However, since the patent of rituximab has expired, there is no interest of the pharmaceutical industry in promoting randomized trials [174].

## Concluding Remarks

This review continues previous publications by our group on failed and interrupted MS trials [8–15] with particular focus on mAbs. We here describe mAbs that failed in relapsing and progressive MS since 2015. On the other side, ocrelizumab and ofatumumab were approved for treatment of MS in this period and another mAb (ublituximab) is in the ‘starting blocks’ for FDA approval.

What conclusions can we draw from these examples? The story of daclizumab demonstrates that mAbs that have been proven to be safe in other autoimmune conditions will not have the same safety profile in MS due to differences in their immunopathogenetic background. It highlights the difficulties of detecting rare AEs in clinical trials as well as the importance of phase IV studies of newly approved treatments [4, 46, 63, 64]. Moreover, it stresses that treatment safety and strategies on how to deal with AEs are meanwhile as important as the efficacy of a DMT.

The example of tabalumab illustrates the complex nature of B cell involvement in MS and emphasizes the important role of Bmem in MS immunopathology [73, 78, 79].

The role of IL-17A and pHERV-W in MS pathogenesis could still not be clarified.

We could demonstrated that failure in one trial does not necessarily mean failure of an agent (e.g., temelimab, off-label use of rituximab), or treatment approach in general (failure of tabalumab and development of belimumab; failure of rituximab, and development of ocrelizumab and ofatunumab).

Moreover, positive preclinical studies do not guarantee the success of an agent in clinical trials (e.g., Vatelizumab). Current animal models are useful tools for developing pharmacological agents but are not able to completely mimic the complexity of human disease. Future mAbs that influence the migration process of immune cells into the inflamed CNS tissue should be as efficacious as natalizumab but without having the side effects of PML.

The failed trials of opicinumab clearly illustrate the challenges of clinical studies on remyelination-promoting therapies and our incomplete understanding of the mechanisms underlying CNS damage and failure of regeneration and repair in MS [198]. The success of future trials on remyelination-promoting therapies will primarily depend on the chosen readout for ‘proof of concept’ (e.g., measuring the P100 latency in a white matter tract as the optic nerve by using FF-VEP), the time of treatment initiation (directly after manifestation of first symptoms), and the appropriate selection of patients (younger patients with clinically isolated syndrome or RRMS) [145, 152]. Moreover, the example of opicinumab also points to the limits of remyelination-promoting therapies to restore functional relevant gray matter lesion and their associated complex neuronal networks [153].

Furthermore, we demonstrated that a sufficiently long trial duration and multicomponent outcome measures with focus on shorter tract-based pathway functions are important for the success of clinical studies in progressive MS, while a large cohort of patients is also necessary for trials with several treatment arms in relapsing–remitting and progressive MS.

The failed trials of natalizumab and rituximab in progressive MS illustrate that effective therapies in relapsing–remitting MS need not necessarily work in progressive MS. The pathophysiological mechanisms of long-standing progressive MS without inflammatory components of relapses and/or GELs in MRI are independent of leukocyte ‘outside-in’ traffic, while this feature is the most relevant component for driving relapses and RAW. Therefore, mAbs for progressive MS must be able to sufficiently cross the blood–brain barrier.

The example of rituximab illustrates very well that its failed approval for MS treatment does not necessarily mean the failure of rituximab in general. In the light of promising results obtained from clinical trials and real-world data and its lower price compared to other approved DMT, it is one of the most commonly used DMT in MS in several countries [187, 194, 195] whose regulatory approval is demanded more and more [188]. However, it is also important to note that the evidence on SAEs of rituximab for MS is very weak [192] and that large randomized controlled trials, head-to-head studies, and long-term safety assessments are urgently needed to provide accurate safety information of rituximab in MS and establish global standards of care [196, 197].

Nonetheless, all mentioned examples of failed mAbs are highly important because they add to our growing understanding of MS pathology and provide valuable information how future studies and outcome measures should be designed. Therefore, their publication is indispensable as otherwise precious information may be lost.

## Supplementary Information

Below is the link to the electronic supplementary material.

Supplementary file1 (PDF 3306 KB)
